# Intravenous paracetamol does not have significant opioid‐sparing effects when used as part of a multimodal analgesic protocol in dogs undergoing elective orthopaedic surgery

**DOI:** 10.1002/vetr.70499

**Published:** 2026-03-13

**Authors:** Robin Stallard, Julia Deutsch, Joanna Murrell

**Affiliations:** ^1^ Highcroft Veterinary Referrals Bristol UK; ^2^ Bristol Vet Specialists Severn Beach UK; ^3^ Small Animal Hospital, Langford Veterinary Services Langford UK

## Abstract

**Background:**

Data evaluating paracetamol combined with NSAIDs in dogs are scarce. Results of clinical studies in dogs investigating intravenous paracetamol vary.

**Methods:**

Dogs were randomised to either receive 10 mg/kg paracetamol intravenously after induction of anaesthesia and every 8 hours during hospitalisation (test) or not (control). In both groups, meloxicam or robenacoxib was administered at the licensed dose, interval and route. Intraoperative nociception or postoperative pain, defined by a Glasgow composite measure pain scale—short form score greater than 4 of 20, was treated with 0.1 mg/kg methadone intravenously as needed.

**Results:**

Data from 14 dogs that received paracetamol and 13 dogs that did not were analysed. There were no statistically significant differences in clinical or demographic data between the two groups. Median (range) rescue methadone requirements were 0.0 (0.0‒0.2) mg/kg and 0.1 (0.0‒0.3) mg/kg for the test and control groups, respectively (*p* = 0.17), with no difference intraoperatively (*p* = 0.72) or postoperatively (*p* = 0.24). Four test and seven control dogs required rescue analgesia perioperatively (*p* = 0.17).

**Limitations:**

Low analgesia requirements in both groups may have resulted in a type two statistical error.

**Conclusion:**

When used as part of a multimodal analgesic protocol, paracetamol did not provide significant opioid‐sparing effects in the perioperative period.

## INTRODUCTION

Paracetamol is commonly used as an analgesic in human medicine^1^ in both oral and intravenous forms. In a meta‐analysis of human pain guidelines, paracetamol was recommended or conditionally recommended in 16 out of 18 published guidelines for the management of common pain conditions.^2^

The World Small Animal Veterinary Association Global Pain Management Guidelines suggest including paracetamol postoperatively as part of a multimodal analgesic protocol for a wide variety of surgical procedures in dogs.^3^ Few adverse effects have been reported with clinically used doses of paracetamol in dogs,^4^, ^5^, ^6^, ^7^, ^8^ making it an attractive analgesic for acute and chronic pain relief.

Despite the common use of paracetamol in humans and dogs, its mechanism of action remains poorly understood. One proposed mechanism of action, supported by experimental evidence, is that paracetamol affects cyclo‐oxygenase (COX) enzymes.^9^ However, the precise nature and clinical significance of this interaction in vivo remain unknown. Other evidence indicates that paracetamol has central effects on CB1 cannabinoid receptors, transient receptor potential vanilloid 1 (TRPV1) channels and serotonin‐related (5‐HT) central pathways.^10^ It is therefore likely that paracetamol targets the pain pathway at multiple sites.

Several studies have investigated the analgesic efficacy of paracetamol in dogs, with variable results. A paracetamol/codeine combination has market authorisation for use in dogs in the UK (Pardale‐V, Dechra). This has been demonstrated to be non‐inferior to meloxicam in dogs undergoing soft tissue and orthopaedic surgery, suggesting an analgesic effect of this combination.^4^ Clinical studies regarding the analgesic efficacy of codeine in dogs are currently lacking. Metabolism of codeine in dogs is comparable with that in people classified as non‐responders to treatment, so it is unlikely to provide significant analgesic benefit in this formulation compared to paracetamol alone.^11^ In dogs undergoing ovariohysterectomy, 15 mg/kg paracetamol intravenously (IV) every 8 hours showed equivalent gradual reduction in postoperative pain when compared to 0.2 mg/kg meloxicam IV or 4 mg/kg carprofen IV administered every 24 hours when all treatments were administered preoperatively and postoperatively.^7^ In contrast, 20 mg/kg paracetamol IV was not associated with a minimal alveolar‐sparing effect when dogs were subjected to a noxious stimulus.^6^ When administered post‐ovariohysterectomy in dogs, this dose showed no detectable analgesic effect compared to a placebo.^5^

NSAIDs are widely used in both veterinary and human medicine and are considered first‐line analgesic agents in the management of postoperative pain.^3^ NSAIDs provide analgesia via inhibition of COX enzymes, resulting in decreased production of prostaglandins,^12^ which act both peripherally and centrally through several mechanisms.^13^, ^14^ These effects include sensitisation of nociceptors and nociceptive neurons to noxious stimuli and upregulation of pathways within the central nervous system that are involved with pain transmission.^15^

NSAIDs have been extensively studied in dogs, and it is broadly accepted that they provide significant analgesia in a variety of scenarios and pain models.^7^, ^16^, ^17^, ^18^, ^19^, ^20^, ^21^, ^22^, ^23^ However, data are scarce regarding the analgesic efficacy of paracetamol in combination with NSAIDs in dogs. One prospective study in dogs undergoing hemilaminectomy demonstrated a reduction in postoperative rescue analgesia when paracetamol was included in the analgesia protocol compared to a placebo group, although there was no difference in intraoperative rescue analgesia requirement.^24^ Paracetamol combined with a range of NSAIDs was found to have synergistic effects on an acute pain model in mice.^25^

The aim of this study was to investigate whether paracetamol combined with an NSAID had a significant perioperative opioid‐sparing effect compared to an NSAID alone in healthy dogs undergoing orthopaedic surgery. The hypothesis was that paracetamol in combination with an NSAID would be associated with a significant perioperative opioid‐sparing effect compared to an NSAID alone.

## MATERIALS AND METHODS

This was a randomised, partially blinded, controlled, multicentre clinical study. The study was approved by both the CVS ethics committee (number CVS‐2023‐009) and the University of Bristol's Animal Welfare and Ethical Review Body (VIN‐24‐009). An Animal Test Certificate—type S was granted by the Veterinary Medicines Directorate (55944/0003) to allow random allocation of dogs to the respective treatment groups. Informed verbal or written owner consent was obtained and recorded.

Dogs presented for unilateral tibial plateau levelling osteotomy (TPLO) and stifle arthrotomy at three referral hospitals (Highcroft Veterinary Referrals [HVR], Bristol Veterinary Specialists [BVS] and Langford Veterinary Services [LVS]) were assessed for eligibility by European board‐certified specialists in veterinary surgery and anaesthesia and analgesia.

Inclusion criteria comprised dogs of American Society of Anaesthesiologists’ classification I or II with a calm enough temperament to facilitate regular pain scoring without causing undue stress. Exclusion criteria included a prior history of surgery on the affected limb, requirement for an arthroscopy during the surgery, previous adverse reactions associated with any of the medications included in the protocol or the presence of a heart murmur or other cardiovascular abnormalities detected on clinical examination.

In dogs that were receiving NSAIDs at the time of admission, the treatment was continued in line with licensed recommendations. Other analgesic medications, such as paracetamol or gabapentin, were discontinued at least 12 hours prior to surgery to minimise their impact on rescue analgesia requirements. Dogs were regularly assessed for pain during their preoperative hospitalisation period by surgeons, anaesthetists and inpatient care teams, and none required additional analgesia during this period. The method of pain assessment was not standardised prior to surgery.

Dogs were randomly assigned to receive 10 mg/kg paracetamol (paracetamol 10 mg/mL solution for infusion, B. Braun Melsungen) IV every 8 hours starting immediately after induction of anaesthesia (test group) or no paracetamol (control group). Paracetamol was administered by hand over 5 minutes. No placebo was used in the control group. An online sequence generator^26^ was used to randomise treatment group by the primary investigator. Odd numbers were allocated to the control group, and even numbers were allocated to the test group. This information was stored on a spreadsheet (Microsoft Excel, Microsoft Corporation) and shared with relevant anaesthesia personnel at each centre to allow appropriate group allocation by the anaesthesia teams as cases were enrolled.

All dogs in both groups received an NSAID at the licensed dose and interval for the duration of their stay in the hospital. This was either meloxicam or robenacoxib. For meloxicam, 0.2 mg/kg meloxicam IV was given as a loading dose followed by 0.1 mg/kg IV or per os every 24 hours (HVR/BVS: Meloxaid, EU Pharmaceuticals; LVS: Metacam, Boehringer). If the meloxicam was a continuation of a previous treatment regimen then 0.1 mg/kg was administered either IV or per os 24 hours after the previous dose. For robenacoxib, 2 mg/kg robenacoxib (Onsior, Elanco UK) was administered subcutaneously after induction of anaesthesia or in recovery and every 24 hours thereafter while hospitalised.

A standardised anaesthetic protocol was used. After premedication with 5 µg/kg dexmedetomidine (Dexdomitor, Vetoquinol) IV and 0.3 mg/kg methadone (HVR/BVS: Comfortan, Dechra; LVS: Methadyne, Zoetis) IV anaesthesia was induced with propofol (PropoFlo Plus, Zoetis) IV to effect to facilitate endotracheal intubation with an appropriately sized cuffed endotracheal tube. Anaesthesia was maintained with isoflurane (HVR/BVS: Isoflurin, Vetpharma Animal Health; LVS: Iso‐Vet, Piramal Critical Care) vaporised in 100% oxygen. The anaesthetic vaporiser was adjusted by the anaesthetist to maintain an adequate plane of surgical anaesthesia, determined by a ventromedial eye position and loss of palpebral reflex and jaw tone. Dogs were allowed to breathe spontaneously through the anaesthetic. If hypercapnia occurred (partial pressure of expired CO_2_ >60 mmHg), this was managed at the discretion of the supervising anaesthetist by optimising depth of anaesthesia and using mechanical ventilation as deemed appropriate.

Heart rate, respiratory rate, pulse oximetry, partial pressure of expired carbon dioxide (etCO_2_), end‐tidal isoflurane concentration, oscillometric systolic, mean and diastolic blood pressures, body temperature and spirometry were continuously monitored throughout anaesthesia with a multiparameter monitor (HVR/LVS: Datex Ohmeda anaesthesia monitor, GE Healthcare; BVS: Mindray ePM 12M Vet, Shenzhen Mindray Animal Medical Technology Co.) and recorded every 5 minutes.

A femoral nerve block was performed with either an electrical nerve stimulator‐guided lateral pre‐iliac approach or an ultrasound‐guided psoas compartment block.^27^ A sciatic nerve block was performed using a lateral approach with either electrical nerve stimulation or ultrasound guidance.^27^ Where one nerve was located using one technique and the second with the other technique, this was recorded as a ‘combined’ approach. Bupivacaine 0.5% (Marcain Polyamp Steripack, Aspen Pharma Trading; Bupivacaine Hydrochloride, ADVANZ Pharma) 0.2 mL/kg was administered per site. Failure of the nerve block was detected subjectively by the supervising anaesthetist if the dog showed significantly higher than expected requirements for rescue analgesia during surgery. Dogs with perceived failure of the nerve block were removed from the study and their analgesia management was determined by a supervising anaesthetist.

Hartmann's solution (Hartmann's Lactated Ringers Solution, B. Braun Melsungen) was infused IV throughout the surgery at 4 mL/kg/h. Prior to surgery, 20 mg/kg cefuroxime IV was administered and continued every 90 minutes until the end of surgery and every 8 hours after surgery until discharge.

Rescue analgesia was administered intraoperatively in response to changes in blood pressure, heart rate and respiratory rate when depth of anaesthesia appeared to be adequate. Increases of approximately 20% in these parameters were used as this reflects common clinical practice in all participating centres, although this was not standardised and the anaesthetist was aware of treatment group. Anaesthetic complications were recorded if they occurred at any point from the time of premedication to the time the dog was returned to its kennel and were managed as deemed appropriate by the supervising anaesthetist.

A unilateral TPLO and arthrotomy were performed as previously described.^28^ All surgeons performing the surgery were experienced and used the same surgical technique. The time from induction of anaesthesia to first incision and total surgical time were recorded to allow comparisons between groups.

Postoperatively, dogs received 2 mL/kg/h Hartmann's solution IV until they were eating and drinking consistently. Pain was assessed every 2 hours postoperatively, from extubation until discharge, by trained and experienced Registered Veterinary Nurses who were blinded to the treatment group using the Glasgow composite measure pain scale—short form (GCMPS‐SF). Section B was not included in these assessments as the motor blockade from the nerve block would have influenced the results for this part of the assessment. If the pain score exceeded 4 of 20,^29^ 0.1 mg/kg methadone IV was administered as rescue analgesia and the pain score was repeated 30 minutes later. This process was repeated until the pain score was 4 of 20 or lower.

Every 8 hours, a Registered Veterinary Nurse not involved with pain assessment would either administer or pretend to administer paracetamol IV according to the group allocation in an envelope on the dog's kennel. A box on the hospital sheet labelled ‘Paracetamol Study’ (occurring every 8 hours) was ticked once this task was completed.

Dogs were discharged at the discretion of the supervising surgeon and anaesthetist once they were satisfied that there was no further requirement for rescue analgesia. This was typically at least 24 hours after surgery. This assessment was based on evaluation of current pain level and requirements for rescue analgesia up to that point. The study ceased at the point of discharge and ongoing analgesia was managed by the surgeon. No follow‐up was performed.

### Data analysis

All the statistical analyses were performed using IBM SPSS statistics for Windows (version 29.0.2.0; IBM Corp.). A sample size calculation was performed using unpublished, retrospective data shared by the anaesthesia team at Manchester Vet Specialists (Manchester, UK). These data showed an approximately 50% opioid‐sparing effect in the perioperative period in dogs undergoing unilateral TPLO that received 10 mg/kg paracetamol IV in addition to an NSAID compared to an NSAID‐only group. With a power of 80% and a *p*‐value of 0.05, a required sample size of nine dogs per group was calculated. Due to the multiple confounding factors affecting these retrospective data, the decision was made to increase the sample size to 15 dogs per group.

Continuous data were assessed for normality using a Shapiro‒Wilk test. Due to the primary outcome measure not being normally distributed, a Mann‒Whitney *U* test was used to compare all continuous data between groups. Categorical data were compared between groups using either a chi‐squared test (for a >2 × 2 contingency table) or a Fisher's exact test (for a 2 × 2 contingency table). A *p*‐value of less than 0.05 was considered statistically significant.

## RESULTS

Thirty dogs were enrolled in the study (15 per group) between July 2023 and October 2024. Three of these dogs were excluded from data analysis. One dog developed diarrhoea prohibiting treatment with an NSAID (control), one had an arthroscopy during the surgery (control) and one had a suspected nerve block failure due to high (outlier) requirements for rescue analgesia (test).

No statistically significant differences were identified in any demographic or clinical variables (Tables 1 and 2).

**TABLE 1:** Summary of demographic data for the control and test groups.

Parameter	Control group	Test group	*p*‐value
Age, mean (SD) (years)	6.15 (3.22)	6.51 (3.09)	0.58
Sex (male, male neutered, female, female neutered)	3, 4, 1, 5	2, 2, 5, 5	0.32
Weight, mean (SD) (kg)	18.4 (8.7)	19.2 (13.2)	0.87
Breed (frequency)	Crossbreed (8), Staffordshire Bull Terrier (1), Golden Retriever (1), American Bulldog (1), German Shepherd dog (1), Border Terrier (1)	Crossbreed (5), Yorkshire Terrier (3), Olde English Bulldog (2), Golden Retriever (1), German Shepherd dog (1), Cocker Spaniel (1), Hungarian Vizsla (1)	0.30

**TABLE 2:** Clinical variables for the control and test groups.

Variable	Sub‐group	Control group	Test group	*p*‐value
Study site	HVR	4	2	0.33
	BVS	7	8	
	LVS	2	4	
Nerve block technique	Ultrasound	5	4	0.50
	Nerve stimulation	3	7	
	Combined	3	3	
	Not recorded	2	0	
Time of first NSAID administration (frequency of different NSAIDs)	Before admit	11 (10 meloxicam, 1 robenacoxib)	10 (meloxicam)	0.79 (0.50)
	At induction of anaesthesia	1 (robenacoxib)	1 (robenacoxib)	
	Immediately after surgery	1 (meloxicam)	3 (meloxicam)	

Abbreviations: BVS, Bristol Veterinary Specialists; HVR, Highcroft Veterinary Referrals; LVS, Langford Veterinary Services.

**FIGURE 1:**
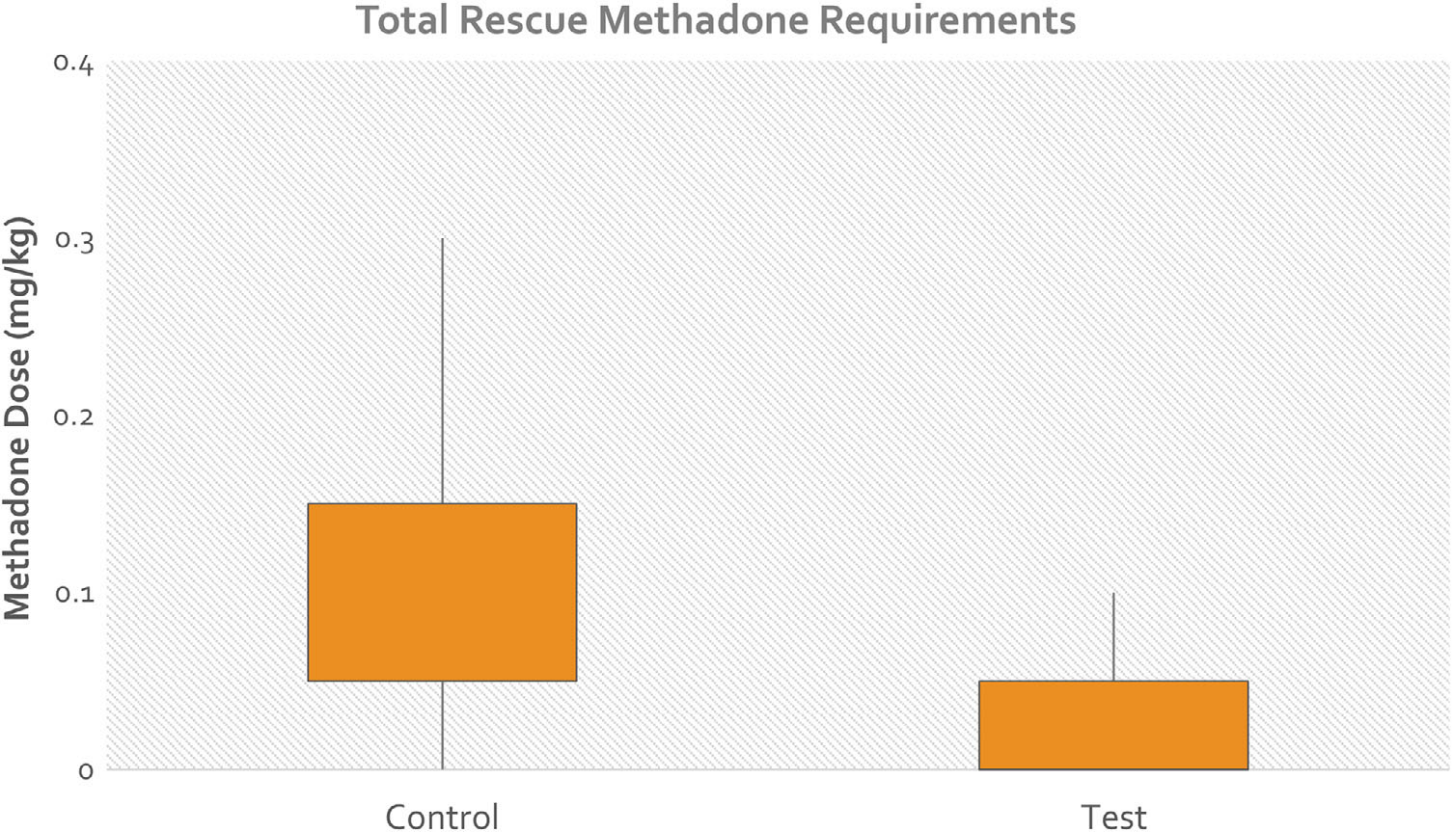
Total rescue methadone requirement in the perioperative period was not statistically significantly different between groups.

**FIGURE 2:**
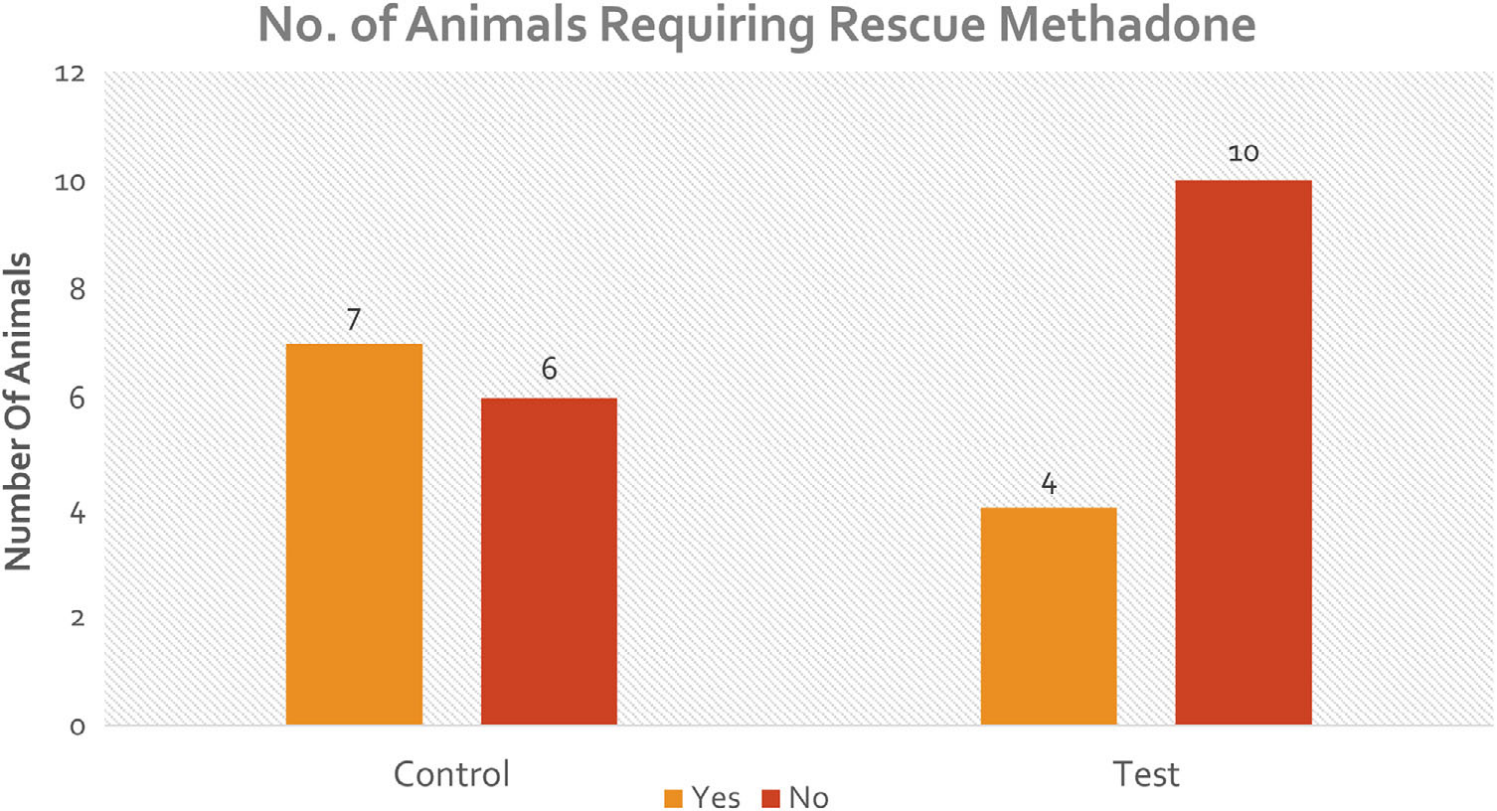
Number of animals requiring rescue methadone at any time point during the study was not statistically significantly different between groups.

There were no significant differences between groups for intraoperative, postoperative or total rescue analgesia requirements (Figures 1 and 2 and Table 3). Intraoperatively, two and three dogs required one rescue methadone dose in the test and control groups, respectively. Postoperatively, two dogs in the test group required one rescue methadone dose, with the remainder needing none. In the control group, three and two dogs required two and one rescue methadone dose, respectively, with the remainder needing none.

**TABLE 3:** Breakdown of the rescue analgesia requirements in the control and test groups throughout the study period and the number of animals requiring rescue analgesia at each time point.

Parameter	Control group	Test group	*p*‐value
Intraoperative rescue, median (range) (mg/kg)	0.0 (0.0‒0.1)	0.0 (0.0‒0.1)	0.72
Number of animals requiring intraoperative rescue	3/13	2/14	0.46
Postoperative rescue, median (range) (mg/kg)	0.0 (0.0‒0.2)	0.0 (0.0‒0.1)	0.24
Number of animals requiring postoperative rescue	5/13	2/14	0.16
Total rescue analgesia, median (range) (mg/kg)	0.1 (0.0‒0.3)	0.0 (0.0‒0.1)	0.17
Total number of animals requiring rescue	7/13	4/14	0.17

Overall, four dogs in the test group required one rescue methadone dose during the perioperative period, with the remainder needing none. In the control group, one, two and four dogs required three, two and one rescue methadone dose, respectively, during the perioperative period, with the remainder needing none.

Only one dog in the control group required both intraoperative and postoperative rescue analgesia, with a total requirement of three rescue methadone doses.

**FIGURE 3:**
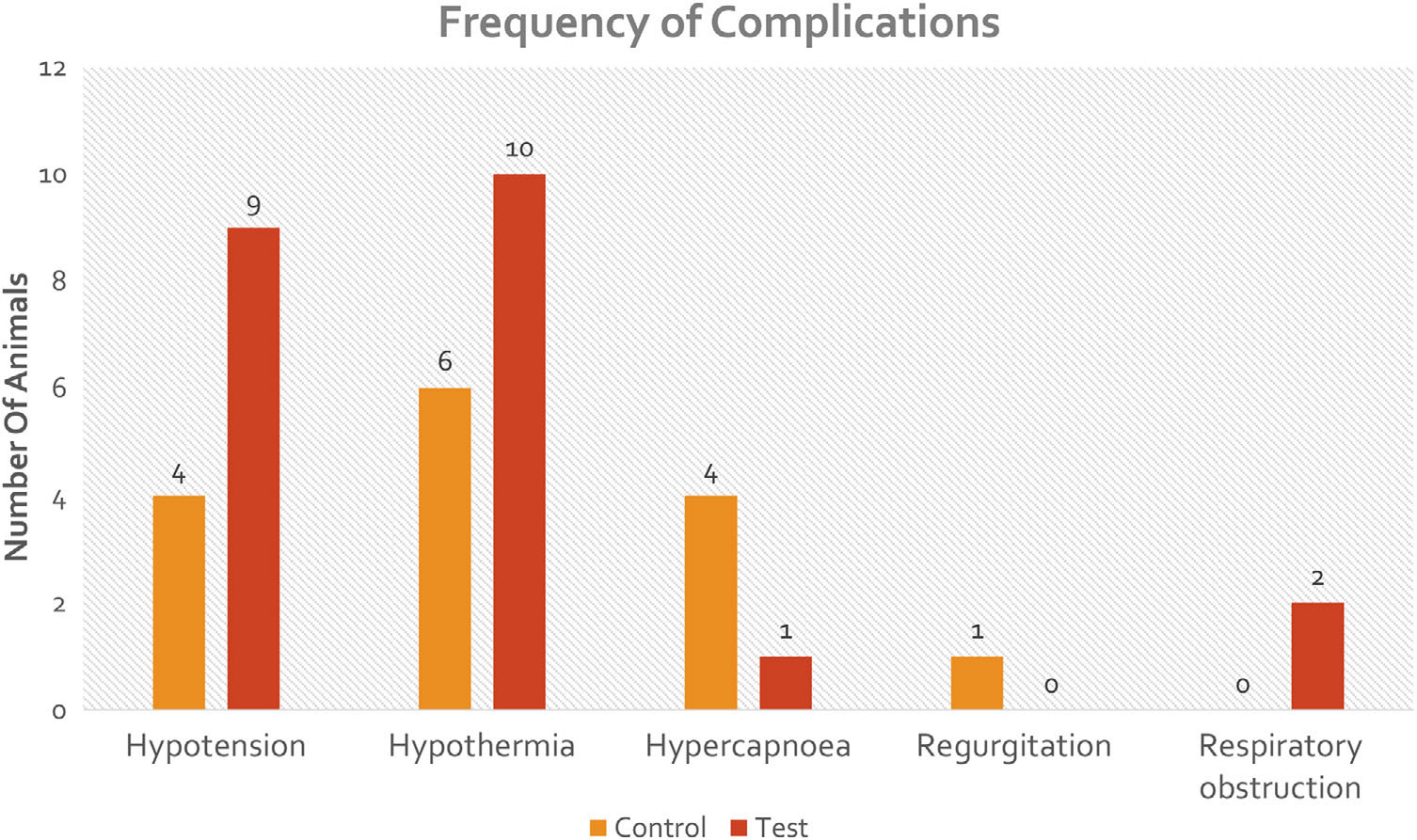
Frequency of anaesthetic complications encountered for control and test groups. No statistically significant differences were found between groups. Definitions: hypothermia—rectal or oesophageal temperature less than 37°C; hypercapnia—end‐tidal carbon dioxide partial pressure greater than 60 mmHg; hypotension—mean arterial blood pressure less than 60 mmHg; regurgitation—stomach contents present in the oral cavity; respiratory obstruction—temporary upper airway obstruction resulting in dyspnoea.

Anaesthetic complications encountered are detailed in Figure 3. The incidence of hypotension in the test group was nine of 14 dogs compared to four of 13 in the control group. This difference was not statistically significant (*p* = 0.09). There was no difference in the total frequency of other anaesthetic complications between the groups (*p* = 0.58).

Where an etCO_2_ of above 60 mmHg, defined in Figure 3 as hypercapnoea, was observed, mechanical ventilation was initiated to maintain etCO_2_ between 35 and 60 mmHg.

The mean (SD) time from induction of anaesthesia to first incision was 84 (17) minutes in the control group and 84 (15) minutes in the test group. The mean (SD) surgical time was 61 (15) minutes in the control group and 71 (21) minutes in the test group. The mean (SD) length of postoperative assessment was 22 (3) hours in the control group and 22 (2) hours in the test group. None of these parameters varied significantly between the two groups.

## DISCUSSION

These data suggest that the addition of paracetamol to a multimodal analgesic protocol is not associated with a significant opioid‐sparing effect in dogs. This would appear to contrast with evidence of synergism between paracetamol and NSAIDs found in other species.^25^ One explanation for this could be that the COX‐inhibiting effects of paracetamol^9^ are insignificant when used in vivo in combination with an NSAID in dogs due to unknown pharmacokinetic and pharmacodynamic differences.

An alternative explanation for this finding could be that the dose and/or dosing interval of paracetamol used in this study was insufficient to confer significant analgesic benefits. A previous pharmacokinetic study showed that plasma concentrations decrease rapidly following IV administration of 10 mg/kg paracetamol, falling below plasma concentrations that are associated with analgesia in people within 60 minutes.^8^ While caution should be exercised when extrapolating analgesic plasma concentrations between species, these data suggest that 10 mg/kg paracetamol IV every 8 hours may be insufficient to provide clinically meaningful analgesia in dogs. However, the dose used in this study is commonly used at the study centres and was the dose used in the data set that determined the sample size.

Two previous clinical studies in dogs used IV paracetamol at higher doses than in the present study.^5^, ^7^ These studies suggest that higher doses of paracetamol are not associated with greater analgesic effects; however, variations in study design and anaesthetic protocols make direct comparisons difficult. Further clinical, pharmacokinetic and pharmacodynamic studies are warranted in this area to ascertain the relationship between dose and analgesic efficacy for IV paracetamol in dogs.

Differences in the efficacy of paracetamol may also be seen with different pain models. Ovariohysterectomy models,^5^, ^7^ where no pre‐existing pain is expected, may yield different results than the orthopaedic model described here, where dogs may have experienced pain in the surgical area prior to intervention. However, it is reasonable to consider a TPLO as a good surgical model for assessment of analgesia. Dogs with cranial cruciate ligament disease are usually mild to moderately painful on the affected limb at presentation and are likely to have a degree of osteoarthritis of the affected joint.^30^ This combination of chronic and acute surgically induced pain make it a reasonable model for orthopaedic‐based analgesic research.

The incidence of hypotension under anaesthesia was evaluated independently of other anaesthetic complications due to the subjective difference between groups and a possible association between intravenous paracetamol administration and a mild and transient reduction in blood pressure in people.^31^ The mechanism responsible for this is uncertain,^32^, ^33^, ^34^ and results of studies in people investigating haemodynamic changes following intravenous paracetamol are inconsistent. Hypotension associated with paracetamol administration in dogs has not previously been described. In this study, the incidence of hypotension was subjectively higher in the group receiving paracetamol; however, this difference was not statistically significant. There are a number of confounding factors in this study that make interpretation of these data difficult, including the use of non‐invasive blood pressure monitoring using multiple monitors, non‐standardised end‐tidal volatile agent partial pressures and a sample size that was not calculated for such an assessment. Further studies using invasive blood pressure monitoring and more appropriate data sets would be required before meaningful conclusions can be made about any such relationship in dogs.

## LIMITATIONS

Several elements of the study design may also have contributed to the lack of observed statistically significant opioid‐sparing effects. When the data were considered descriptively, the addition of paracetamol to the protocol was associated with a greater than 50% drop in both total rescue opioid use and the number of dogs requiring rescue analgesia, but this difference was not statistically significant. This may be the result of chance and random error but could also be an indication of a type two statistical error. However, a sample size calculation was performed with a comparable data set, suggesting that an appropriate sample size was used.

A relatively low number of dogs (41% across both groups) required any rescue analgesia during the perioperative period. This low requirement for rescue analgesia may suggest that the baseline analgesia used in the study design was such that any additional effect from paracetamol was not observable. However, given the well‐documented efficacy of the locoregional techniques used in this study for perioperative analgesia in orthopaedic patients undergoing stifle surgery, it was considered unethical to recruit dogs to this study without including a locoregional block in the study protocol.

The choice of which technique to use for the locoregional block was dependent on the preference of the anaesthetist. This was to ensure the highest possible success rate of the locoregional block as failure would compromise patient care and result in exclusion from the study. Given the similarities in the approach and methodology with these locoregional techniques and the lack of difference in technique used between groups, the authors consider it unlikely to have significantly impacted rescue analgesia requirements.

Two different NSAIDs were used in the study: meloxicam and robenacoxib. This is representative of a clinical caseload where a range of NSAIDs are commonly encountered. A study comparing meloxicam and robenacoxib for postoperative analgesia in dogs undergoing orthopaedic surgery showed similar analgesia between groups.^35^

The lack of standardisation of the time of administration of NSAIDs is a limitation of the study design. The majority of dogs that were presented for surgery were already receiving an NSAID at home and this was continued in line with licensed recommendations during hospitalisation. If this was not the case, the timing of administration was decided by the supervising anaesthetist based on their perception of the risk of developing acute kidney injury that could result from reduced prostaglandin synthesis in the face of anaesthesia‐induced hypotension, combined with hospital protocols at the study centres. It is possible that the timing of administration of the NSAID in relation to the surgery may have had an impact on the requirement for rescue analgesia. However, even when administered on recovery from anaesthesia, it is likely that significant analgesia attributable to the NSAID would be seen by the time of recovery from the sensory blockage from the nerve blocks.^36^, ^37^ This, combined with the lack of difference in NSAID administration timing between groups and low number of patients not receiving it preoperatively, leads the authors to believe this is unlikely to be a source of significant error.

The anaesthetist supervising the anaesthetic was not blinded to the study group, and this may have led to bias in decision making around the requirement for additional intraoperative analgesia. Intraoperative nociception was judged subjectively by the supervising anaesthetist, as outlined previously. In many studies, requirement for rescue analgesia intraoperatively is based on changes in physiological variables (e.g., >20% increase in blood pressure compared to a baseline taken before the start of surgery).^38^, ^39^, ^40^ This was not standardised in the present study. Using standardised criteria for the detection of intraoperative nociception would have strengthened the study methodology. However, the number of dogs requiring intraoperative rescue analgesia was very low and similar between groups. Evaluation of postoperative opioid‐sparing effects may have been easier if fentanyl was used for intraoperative rescue analgesia due to its shorter duration of action compared to methadone.^41^, ^42^ However, the aim of the study was to measure opioid‐sparing effect throughout the perioperative period and the use of multiple opioids may have complicated this analysis. This, coupled with common clinical practice at participating centres, resulted in methadone being used throughout the study.

Variability in anaesthetic depth may have altered the supervising anaesthetists’ ability to detect nociception. Volatile agent vaporiser setting was adjusted to maintain an appropriate depth of anaesthesia in line with common clinical practice, however such titrations were not standardised.

Postoperative pain scores were not carried out by a single assessor as this would not have been practical for overnight assessments in a multicentre study. However, only staff who were trained and experienced in how to carry out pain assessments using the GCMPS‐SF performed the pain scores. The GCMPS‐SF is widely used both clinically and in research work for the evaluation of postoperative pain in patients undergoing orthopaedic surgery.^43^ Inter‐individual variability in assessment of the GCMPS‐SF exists, however excellent agreement has previously been demonstrated between Registered Veterinary Nurses.^44^ Alternative methods of pain assessment were considered, but familiarity and experience are important factors when using any pain score, and the GCMPS‐SF is the most commonly used canine pain score in all participating centres.

A baseline pain score taken prior to surgery may have been a pertinent addition to the dogs’ preoperative assessment, as dogs that were more painful prior to surgery may have had greater postoperative analgesia requirements as has been shown in people.^45^

## CONCLUSION

When added to a multimodal analgesic protocol including locoregional techniques and an NSAID, 10 mg/kg paracetamol IV every 8 hours did not confer significant opioid‐sparing effects in healthy dogs undergoing elective orthopaedic surgery. Furthermore, larger scale studies are warranted to investigate the analgesic efficacy of paracetamol combined with an NSAID, and to investigate the significance of different doses and dosing intervals of intravenous paracetamol in dogs.

## AUTHOR CONTRIBUTIONS

All the authors made significant contributions to the study design, ethical approval, case selection and recruitment, data acquisition and interpretation and manuscript preparation.

## CONFLICT OF INTEREST STATEMENT

The authors declare they have no conflicts of interest.

## FUNDING INFORMATION

The authors received no specific funding for this work.

## ETHICS STATEMENT

None

## Data Availability

Data are available from the corresponding author upon reasonable request.

## References

[1] Moore RA , Moore N . Paracetamol and pain: the kiloton problem. Eur J Hosp Pharm. 2016;23:187‒188. 31156845 10.1136/ejhpharm-2016-000952 PMC6451482

[2] Freo U , Ruocco C , Valerio A , Scagnol I , Nisoli E . Paracetamol: a review of guideline recommendations. J Clin Med. 2021;10(15):3420. 34362203 10.3390/jcm10153420 PMC8347233

[3] Monteiro BP , Lascelles BDX , Murrell J , Robertson S , Steagall PVM , Wright B . 2022 WSAVA guidelines for the recognition, assessment and treatment of pain. J Small Anim Pract. 2023;64:177‒254.

[4] Pacheco M , Knowles TG , Hunt J , Slingsby LS , Taylor PM , Murrell JC . Comparing paracetamol/codeine and meloxicam for postoperative analgesia in dogs: a non‐inferiority trial. Vet Rec. 2020;187:e61. 31900324 10.1136/vr.105487

[5] Leung J , Beths T , Carter JE , Munn R , Whittem T , Bauquier SH . Intravenous acetaminophen does not provide adequate postoperative analgesia in dogs following ovariohysterectomy. Animals. 2021;11:3609. 34944384 10.3390/ani11123609 PMC8697971

[6] González‐Blanco P , Canfrán S , Mota R , de Segura IAG , Aguado D . Effects of a single paracetamol injection on the sevoflurane minimum alveolar concentration in dogs. Can J Vet Res. 2020;84:37‒43. 31949328 PMC6921988

[7] Hernández‐Avalos I , Valverde A , Ibancovichi‐Camarillo JA , Sánchez‐Aparicio P , Recillas‐Morales S , Osorio‐Avalos J , et al. Clinical evaluation of postoperative analgesia, cardiorespiratory parameters and changes in liver and renal function tests of paracetamol compared to meloxicam and carprofen in dogs undergoing ovariohysterectomy. PLoS One. 2020;15(2):e0223697. 32059002 10.1371/journal.pone.0223697 PMC7021320

[8] Serrano‐Rodríguez JM , Mengual C , Quirós‐Carmona S , Fernández J , Domínguez JM , Serrano‐Caballero JM , et al. Comparative pharmacokinetics and a clinical laboratory evaluation of intravenous acetaminophen in beagle and Galgo Español dogs. Vet Anaesth Analg. 2018;46(2):226–235. 30713054 10.1016/j.vaa.2018.09.042

[9] Lucas R , Warner TD , Vojnovic I , Mitchell JA . Cellular mechanisms of acetaminophen: role of cyclo‐oxygenase. FASEB J. 2005;19:1‒15. 15705740 10.1096/fj.04-2437fje

[10] Ohashi N , Kohno T . Analgesic effect of acetaminophen: a review of known and novel mechanisms of action. Front Pharmacol. 2020;11:580289. 33328986 10.3389/fphar.2020.580289 PMC7734311

[11] KuKanich B . Pharmacokinetics of acetaminophen, codeine, and the codeine metabolites morphine and codeine‐6‐glucuronide in healthy Greyhound dogs. J Vet Pharmacol Ther. 2010;33:15‒21. 20444020 10.1111/j.1365-2885.2009.01098.x PMC2867071

[12] Lees P , Landoni MF , Giraudel J , Toutain PL . Pharmacodynamics and pharmacokinetics of nonsteroidal anti‐inflammatory drugs in species of veterinary interest. J Vet Pharmacol Ther. 2004;27:479‒490. 15601442 10.1111/j.1365-2885.2004.00617.x

[13] Funk CD . Prostaglandins and leukotrienes: advances in eicosanoid biology. Science. 2001;294:1871‒1875. 11729303 10.1126/science.294.5548.1871

[14] Ricciotti E , FitzGerald GA . Prostaglandins and inflammation. Arterioscler Thromb Vasc Biol. 2011;31(5):986‒1000. 21508345 10.1161/ATVBAHA.110.207449 PMC3081099

[15] Burian M , Geisslinger G . COX‐dependent mechanisms involved in the antinociceptive action of NSAIDs at central and peripheral sites. Pharmacol Ther. 2005;107(2):139‒154. 15993252 10.1016/j.pharmthera.2005.02.004

[16] Nell T , Bergman J , Hoeijmakers M , Van Laar P , Horspool LJI . Comparison of vedaprofen and meloxicam in dogs with musculoskeletal pain and inflammation. J Small Anim Pract. 2002;43(5):208‒212. 12038853 10.1111/j.1748-5827.2002.tb00059.x

[17] Cassemiche A , Schoffit S , Manassero M , Kohlhauer M . Comparison of grapiprant and meloxicam for management of postoperative joint pain in dogs: a randomized, double‐blinded, prospective clinical trial. J Vet Intern Med. 2024;38(4):2324‒2332. 38944675 10.1111/jvim.17136 PMC11256200

[18] Tomacheuski R , Taffarel M , Cardoso G , Derussi A , Ferrante M , Volpato R , et al. Postoperative analgesic effects of laserpuncture and meloxicam in bitches submitted to ovariohysterectomy. Vet Sci. 2020;7(3):94. 32708066 10.3390/vetsci7030094 PMC7559566

[19] Tsai T‐Y , Chang SK , Chou P‐Y , Yeh L‐S . Comparison of postoperative effects between lidocaine infusion, meloxicam, and their combination in dogs undergoing ovariohysterectomy. Vet Anaesth Analg. 2013;40(6):615‒622. 23837712 10.1111/vaa.12064

[20] Laredo FG , Belda E , Murciano J , Escobar M , Navarro A , Robinson KJ , et al. Comparison of the analgesic effects of meloxicam and carprofen administered preoperatively to dogs undergoing orthopaedic surgery. Vet Rec. 2004;155(21):667‒671. 15581141 10.1136/vr.155.21.667

[21] Schmid VB , Spreng DE , Seewald W , Jung M , Lees P , King JN . Analgesic and anti‐inflammatory actions of robenacoxib in acute joint inflammation in dog. J Vet Pharmacol Ther. 2010;33(2):118‒131. 20444036 10.1111/j.1365-2885.2009.01117.x

[22] Bustamante R , Daza MA , Canfrán S , García P , Suárez M , Trobo I , et al. Comparison of the postoperative analgesic effects of cimicoxib, buprenorphine and their combination in healthy dogs undergoing ovariohysterectomy. Vet Anaesth Analg. 2018;45(4):545‒556. 29716837 10.1016/j.vaa.2018.01.003

[23] Laird JMA , Herrero JF , Garcia De La Rubia P , Cervero F . Analgesic activity of the novel COX‐2 preferring NSAID, meloxicam in mono‐arthritic rats: central and peripheral components. Inflamm Res. 1997;46(6):203‒210. 9243303 10.1007/s000110050174

[24] Burger NC , Bosmans T , Bhatti SFM , Ooms S , Broeckx BJG , Polis I , et al. Paracetamol add‐on treatment for perioperative pain management in dogs undergoing single‐site thoracolumbar hemilaminectomy: a prospective clinical study. Vlaams Diergeneeskundig Tijdschrift. 2023;92:305‒313.

[25] Miranda HF , Puig MM , Prieto JC , Pinardi G . Synergism between paracetamol and nonsteroidal anti‐inflammatory drugs in experimental acute pain. Pain. 2006;121(1‒2):22‒28. 16480830 10.1016/j.pain.2005.11.012

[26] Randomness and Integrity Services. RANDOM.ORG. 2009. Available from: www.random.org/lists/. Accessed 28 Jun 2023.

[27] Portela DA , Verdier N , Otero PE . Regional anesthetic techniques for the pelvic limb and abdominal wall in small animals: a review of the literature and technique description. Vet J. 2018;238:27‒40. 30103913 10.1016/j.tvjl.2018.07.003

[28] Slocum B , Slocum TD . Tibial plateau leveling osteotomy for repair of cranial cruciate ligament rupture in the canine. Vet Clin North Am: Small Anim Pract. 1993;23(4):777‒795. 8337790 10.1016/s0195-5616(93)50082-7

[29] Reid J , Nolan Am , Hughes J , Lascelles D , Pawson P , Scott E . Development of the short‐form Glasgow composite measure pain scale (CMPS‐SF) and derivation of an analgesic intervention score. Anim Welfare. 2007;16(S1):97‒104.

[30] Pegram C , Diaz‐Ordaz K , Brodbelt DC , Chang Y‐M , von Hekkel AF , Wu C‐H , et al. Target trial emulation: does surgical versus non‐surgical management of cranial cruciate ligament rupture in dogs cause different outcomes? Prev Vet Med. 2024;226:106165. 38503655 10.1016/j.prevetmed.2024.106165

[31] Maxwell EN , Johnson B , Cammilleri J , Ferreira JA . Intravenous acetaminophen‐induced hypotension: a review of the current literature. Ann Pharmacother. 2019;53(10):1033‒1041. 31046402 10.1177/1060028019849716

[32] Krajčová A , Matoušek V , Duška F . Mechanism of paracetamol‐induced hypotension in critically ill patients: a prospective observational cross‐over study. Aust Crit Care. 2013;26(3):136‒141. 22424816 10.1016/j.aucc.2012.02.002

[33] Dannesboe J , Bastrup JA , Nielsen KH , Munck P , Thomsen MB , Hawkins CL , et al. Paracetamol metabolism by endothelial cells—potential mechanism underlying intravenous paracetamol‐induced hypotension. Pharmacol Res. 2025;211:107540. 39653302 10.1016/j.phrs.2024.107540

[34] Van Der Horst J , Manville RW , Hayes K , Thomsen MB , Abbott GW , Jepps TA . Acetaminophen (paracetamol) metabolites induce vasodilation and hypotension by activating Kv7 potassium channels directly and indirectly. Arterioscler Thromb Vasc Biol. 2020;40(5):1207‒1219. 32188278 10.1161/ATVBAHA.120.313997 PMC7180128

[35] Gruet P , Seewald W , King JN . Evaluation of subcutaneous and oral administration of robenacoxib and meloxicam for the treatment of acute pain and inflammation associated with orthopedic surgery in dogs. Am J Vet Res. 2011;72(2):184‒193. 21281192 10.2460/ajvr.72.2.184

[36] Borer LR , Peel JE , Seewald W , Schawalder P , Spreng DE . Effect of carprofen, etodolac, meloxicam, or butorphanol in dogs with induced acute synovitis. Am J Vet Res. 2003;64(11):1429‒1437. 14620781 10.2460/ajvr.2003.64.1429

[37] Cathasaigh MO , Read MR , Atilla A , Schiller T , Kwong GPS . Blood concentration of bupivacaine and duration of sensory and motor block following ultrasound‐guided femoral and sciatic nerve blocks in dogs. PLoS One. 2018;13(3):e0193400. 29505566 10.1371/journal.pone.0193400 PMC5837095

[38] Didier C , Faucher S , Ferrer MS , Lapouge M , Junot S , Jourdan G . Postoperative opioid‐free analgesia in dogs undergoing tibial plateau leveling osteotomy: a feasibility study. Front Vet Sci. 2024;11:1394366. 39036794 10.3389/fvets.2024.1394366 PMC11257878

[39] Lovell S , Simon B , Boudreau EC , Mankin J , Jeffery N . Randomized clinical trial comparing outcomes after fentanyl or ketamine‐dexmedetomidine analgesia in thoracolumbar spinal surgery in dogs. J Vet Intern Med. 2022;36(5):1742‒1751. 35962706 10.1111/jvim.16514 PMC9511085

[40] Adami C , Veres‐Nyéki K , Spadavecchia C , Rytz U , Bergadano A . Evaluation of peri‐operative epidural analgesia with ropivacaine, ropivacaine and sufentanil, and ropivacaine, sufentanil and epinephrine in isoflurane anesthetized dogs undergoing tibial plateau levelling osteotomy. Vet J. 2012;194(2):229‒234. 22658249 10.1016/j.tvjl.2012.04.019

[41] Sano T , Nishimura R , Kanazawa H , Igarashi E , Nagata Y , Mochizuki M , et al. Pharmacokinetics of fentanyl after single intravenous injection and constant rate infusion in dogs. Vet Anaesth Analg. 2006;33(4):266‒273. 16764592 10.1111/j.1467-2995.2005.00266.x

[42] Ingvast‐Larsson C , Holgersson A , Bondesson U , Lagerstedt A‐S , Olsson K . Clinical pharmacology of methadone in dogs. Vet Anaesth Analg. 2010;37(1):48‒56. 20017819 10.1111/j.1467-2995.2009.00476.x

[43] Testa B , Reid J , Scott ME , Murison PJ , Bell AM . The short form of the Glasgow composite measure pain scale in post‐operative analgesia studies in dogs: a scoping review. Front Vet Sci. 2021;8:751949. 34660773 10.3389/fvets.2021.751949 PMC8515184

[44] Marco‐Martorell M , Duffy N , Martinez M , Maddox T , Robson K . Agreement of pain assessment using the short form of the canine Glasgow composite measure pain scale between veterinary students, veterinary nurses, veterinary surgeons, and ECVAA‐diplomates. Animals. 2024;14(16):2310. 39199844 10.3390/ani14162310 PMC11350858

[45] Wu F , Liu J , Zheng L , Chen C , Basnet D , Zhang J , et al. Preoperative pain sensitivity and its correlation with postoperative acute and chronic pain: a systematic review and meta‐analysis. Br J Anaesth. 2024;133(3):591‒604. 38879440 10.1016/j.bja.2024.05.010

